# Expression and Regulatory Network Analysis of BICC1 for Aged Sca-1-Positive Bone Narrow Mesenchymal Stem Cells

**DOI:** 10.1155/2022/4759172

**Published:** 2022-06-15

**Authors:** Zhongshuang Liu, Chuntao Ou, Siyu Xiang, Qi Xie, Shuangjiang Che, Dandi Zhang, Lingna Meng, Ziqi Liu, Guohong Zhang, Ying Wang, Yun Huang, Wenjing Zhang, Yongqiang Deng

**Affiliations:** ^1^Department of Stomatology, Shenzhen University General Hospital, Shenzhen University, Shenzhen, 518055 Guangdong, China; ^2^Institute of Stomatological Research, Shenzhen University, Shenzhen, 518055 Guangdong, China; ^3^Department of Stomatology, Shenzhen University, Shenzhen, 518000 Guangdong, China; ^4^Department of Stomatology, Harbin Children's Hospital, Harbin, 150001 Heilongjiang, China; ^5^Department of Laboratory Diagnosis, The Second Affiliated Hospital of Harbin Medical University, Harbin, 150001 Heilongjiang, China

## Abstract

**Background:**

The impaired osteoblastic differentiation of bone marrow mesenchymal stem cells (BMSCs) is a major cause of bone remodeling imbalance and osteoporosis. The bicaudal C homologue 1 (BICC1) gene is a genetic regulator of bone mineral density (BMD) and promotes osteoblast differentiation. The purpose of this study is to explore the probable function of BICC1 in osteoporosis and osteogenic differentiation of aged BMSCs.

**Methods:**

We examined the GSE116925 microarray dataset obtained from the Gene Expression Omnibus (GEO) database. The GEO2R algorithm identified differentially expressed genes (DEGs) in Sca-1+ BMSCs from young (3 months old) and old (18 months old) mice. Then, to identify the most crucial genes, we used pathway enrichment analysis and a protein-protein interaction (PPI) network. Furthermore, starBase v2.0 was used to generate the regulatory networks between BICC1 and related competing endogenous RNAs (ceRNAs). NetworkAnalyst was used to construct TF-gene networks and TF-miRNA-gene networks of BICC1 and ceRNA. Furthermore, we investigated the *Bicc1* expression in aged Sca-1-positive BMSCs.

**Result:**

We detected 923 DEGs and discovered that epidermal growth factor receptor (EGFR) was the top hub gene with a high degree of linkage. According to the findings of the PPI module analysis, EGFR was mostly engaged in cytokine signaling in immune system and inflammation-related signaling pathways. 282 ceRNAs were found to interact with the BICC1 gene. EGFR was not only identified as a hub gene but also as a BICC1-related ceRNA. Then, we predicted 11 common TF-genes and 7 miRNAs between BICC1 and EGFR. Finally, we found that BICC1 mRNA and EGFR mRNA were significantly overexpressed in aged Sca-1-positive BMSCs.

**Conclusion:**

As a genetic gene that affects bone mineral density, BICC1 may be a new target for clinical treatment of senile osteoporosis by influencing osteogenic differentiation of BMSCs through EGFR-related signaling. However, the application of the results requires support from more experimental data.

## 1. Introduction

The progression of an aging body is one of the most prevalent, yet stubborn, medical challenges. The cause of the majority of age-related illnesses, including those that cause changes in skeletal tissue composition, such as osteoporosis, remains unclear. Osteoporosis is a systemic bone disease characterized by reduced bone mass, altered bone microstructure, and fragility fractures [1]. Age has an important impact on bone loss and fracture. The most noticeable feature of osteoporosis in the elderly is an imbalance in bone remodeling characterized by increased osteoclast absorption and decreased osteoblast bone formation [2]. However, bone loss occurs as a result of aging or certain pathological conditions in which bone resorption exceeds bone formation [3]. Bone marrow mesenchymal stem cells have multidirectional differentiation potential [4]. In the presence of appropriate environmental signals, BMSCs have the ability to undergo strong osteogenic differentiation. Recently, the role of genetic genes in regulating BMSC differentiation through the osteogenic pathway during osteoporosis has attracted much attention [5].

BICC1 is a multicellular animal evolutionarily conserved RNA binding protein that plays a vital role in signal transduction pathways, organ development, and homeostasis [6]. At least three *Biccl* mutant mice (jcpk, bpk, and 67Gso) have been reported. The frequency of humoral cysts in the kidneys increases in these mutants, as does the expansion of the liver ducts and pancreas. These characteristics are extremely similar to those reported in humans with hereditary polycystic kidney disease (PKD) [7]. The *Bicc1* mutation also causes pancreatic developmental abnormalities, including a decrease in insulin-producing cells, which leads to diabetes.

Through comprehensive genetic approaches, Mesner et al. discovered that single nucleotide polymorphisms (SNPs) in *Bicc1* were strongly associated with bone mineral density. The researchers discovered that BMD in *Bicc1*^+/-^ mice was considerably lower than in wild-type mice, demonstrating that *Bicc1* was a genetic predictor of BMD. Further research revealed that BICC1 was coexpressed with PKD2, a protein involved in osteoblast differentiation. In primary cranial osteoblasts, knocking out *Bicc1* or *Pkd2* had an effect on osteoblast differentiation [8]. *Pkd2* overexpression can rescue osteoblast functional deficits induced by *Bicc1* deficiency, indicating that *Bicc1* may control osteoblast differentiation through *Pkd2*. Other research has shown that polycystin-1 (PC1), expressed by the *Pkd1* gene, interacts with polycystin-2 (PC2) to form an interdependent signal complex, and that the phenotype of bone-specific *Pkd1* deficient mice is similar to age-related bone loss. PC1 and PC2 can combine to produce a complex that binds to primary cilia in osteoblasts and functions as a “mechanical sensor” to regulate bone mass [9]. In 2021, the five priority osteoporosis genes were discovered from 38 reported BMD genome-wide association studies (GWAS), with *Bicc1* having the highest Tier-1 SNPs, indicating that *Bicc1* was a crucial gene in controlling osteoporosis [10].

There is currently a lack of comprehensive evaluation of the effect of BICC1 on osteoporosis and osteogenic differentiation of aged BMSCs. Recent data suggests that the research of biological pathways behind various diseases has been facilitated by the discovery of gene maps using bioinformatics analysis. Therefore, we obtained gene expression data of BMSCs isolated from young and aged mice. Based on identified DEGs, we performed gene set enrichment analysis and pathway analysis to better understand the biological process of genome-based expression. The PPI network was used to screen hub genes, and PPI function modules were created to predict the signaling pathways involved in hub genes. The target genes of BICC1 were screened by the ceRNA network, and then, the shared miRNAs and transcription factors of BICC1 and hub genes were screened. We created an mRNA-ceRNA network of BMSCs in elderly osteoporosis, which might lead to new discoveries about the etiology and therapy of osteoporosis.

## 2. Materials and Methods

### 2.1. Data Collection

The dataset GSE116925 was retrieved by screening from the GEO database [11]. GEO is committed to developing a gene expression data warehouse as well as online facilities for retrieving gene expression data from any species or man-made source. GEO mostly contains chip data and a minor amount of sequencing data. Li C et al. donated the microarray file dataset GSE116925. It featured gene expression data from BMSCs isolated from young (3 months old) and aged (18 months old) mice. The population of Sca-1+CD29+CD45–CD11b–BMSCs was sorted for experiments. The dataset is based on the Affymetrix mouse transcript array 1.0 (transcript (gene) CSV version) platform GPL20775 (mta-1 0).

### 2.2. Data Processing and Identification of DEGs

Hub gene research is an essential step in anticipating illness therapeutic targets [12]. We discovered DEGs between BMSCs from young and aged mice using the online analysis tool GEO2R (https://www.ncbi.nlm.nih.gov/geo/geo2r/). Benjamini-Hochberg was applied to the dataset for the control of false discovery rate (FDR) [13], and *Padj* < 0.001 was utilized as the database's cut-off criteria.

### 2.3. GO and KEGG Enrichment Analysis

We used the Database for Annotation, Visualization, and Integrated Discovery (DAVID) (https://david.ncifcrf.gov/tools.jsp) to explore the Kyoto Encyclopedia of Genes and Genes (KEGG) pathway of differentially expressed genes and gene modules of interest. DAVID provides a comprehensive set of functional annotation tools for investigators to understand the biological meaning behind large lists of genes [14]. We ran the Gene Ontology (GO) analysis on the DEGs through WebGestalt [15], and the results are divided into three sections: molecular function, cellular component, and biological process. The cut-off criteria were established at *P* < 0.05 and FDR < 0.25.

### 2.4. Construction of PPI Network and Clustering of Functional Modules

Constructing a PPI network is an essential step in completing the research. PPI network analysis is useful for studying illness molecular mechanisms and discovering new therapeutic targets in a methodical manner [16]. In this article, we uploaded the aforesaid differentially expressed genes to the STRING (http://stringdb.org) online database, with the cut-off criterion set to interaction score > 0.4, in order to construct a PPI network. STRING is a database that allows us to explore and predict the interactions of known proteins [17]. It now contains 24,584,628 proteins from 5,090 organisms. Protein interactions involve both direct physical interactions and indirect functional connections. PPIs were put into Cytoscape [18] for additional analysis in order to mine the core regulatory genes. Cytoscape (https://cytoscape.org/) is an open-source software platform for visualizing molecular interaction networks and biological pathways; it also has a plethora of plugins that provide great convenience for researchers. MCODE is a program that searches for clusters (highly linked sections) in a large gene (or protein) network [19]. This could reveal the primary functional modules of differentially expressed genes.

### 2.5. Identification of Hub Gene

CytoHubba [20] was used to identify hub genes. CytoHubba is a significant app in Cytoscape that can explore important nodes/hubs and fragile motifs in an interactome network using a variety of topological algorithms such as degree, maximum neighborhood component (MNC), and maximal clique centrality (MCC), as well as centralities based on shortest paths such as bottleneck (BN), eccentricity, closeness, radiality, betweenness, and stress. In this study, the top ten nodes ranked by degree were identified as hub genes.

### 2.6. Exploration of Key ceRNA Related to BICC1

starBase v2.0 can identify more than 4.1 million RBP-RNA, 2.9 million miRNA-mRNA, 4.1 million miRNA-ncRNA, and 1.5 million RNA-RNA interactions from multidimensional sequencing data [21]. We designated BICC1 as a ceRNA GENE of interest and found target genes that may have a high level of interaction with it using a *P* < 0.001 and FDR < 0.01 cut-off criterion. Venny 2.1.0 (https://bioinfogp.cnb.csic.es/tools/venny/index.html) was used to find common genes between hub and target genes.

### 2.7. TF-Gene Network

The construction of a TF-gene network helps assess the impact of TF-genes on the gene function pathway and the expression level of important genes. NetworkAnalyst (https://www. networkanalyst.ca/) is a comprehensive web platform for performing gene expression on a wide range of species [22]. The TF-gene network was built using the ChEA database [23], which is part of the NetworkAnalyst platform. ChEA is a database of transcription factor targets derived from combining literature-curated Chip-X data.

### 2.8. Gene-miRNA Network

The association between genes and miRNA was demonstrated in the gene-miRNA network, which will aid in the investigation of the mechanism of gene connection. The gene-miRNA network in NetworkAnalyst is displayed using extensive experimentally validated miRNA-gene interaction data from TarBase v8.0 [24].

## 3. Results

### 3.1. Identification of DEGs

**Figure 1:**
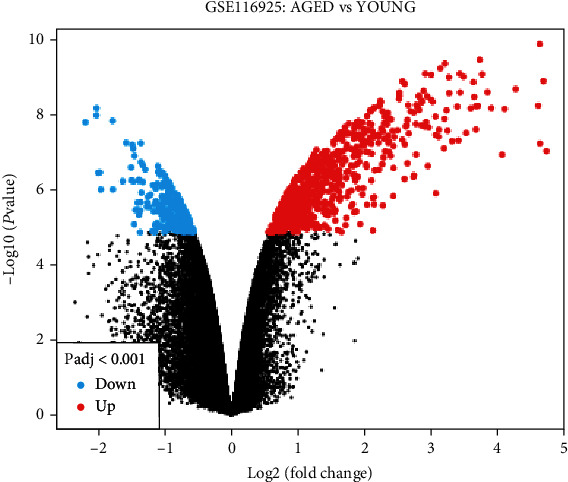
A volcano plot showed the DEGs between aged and young Sca-1+ BMSCs.

Compared to the young Sca-1+ BMSCs, GEO2R identified 923 differently expressed genes in the aged mice group, including 702 upregulated genes and 221 downregulated genes. The cut-off standard was set at *Padj* < 0.001. The volcano map in Figure 1 depicts the end consequence.

### 3.2. GO and KEGG Enrichment Analysis of DEGs

**Figure 2:**
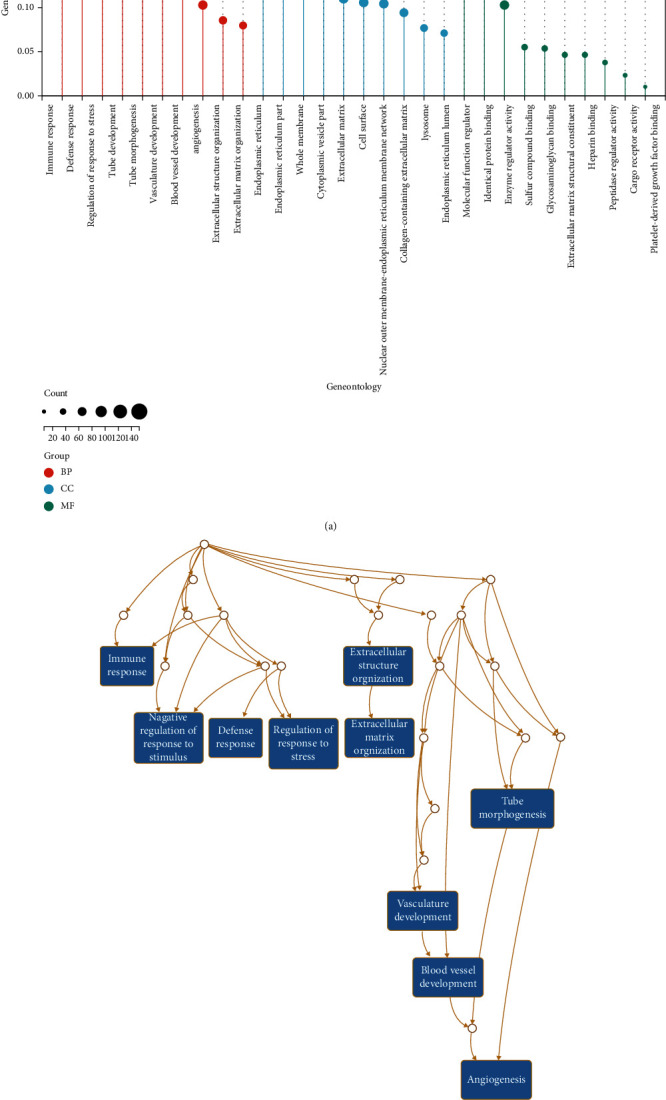
GO enrichment analysis results of DEGs. (a) The lollipop chart showed the top 10 results of BP, MF, and CC. (b) Network displayed the top 10 results, including immune response, negative regulation of response to stimulus, defense response, regulation of response to stress, tube morphogenesis, vasculature development, blood vessel development, angiogenesis, extracellular structure organization, and extracellular matrix organization.

**Figure 3:**
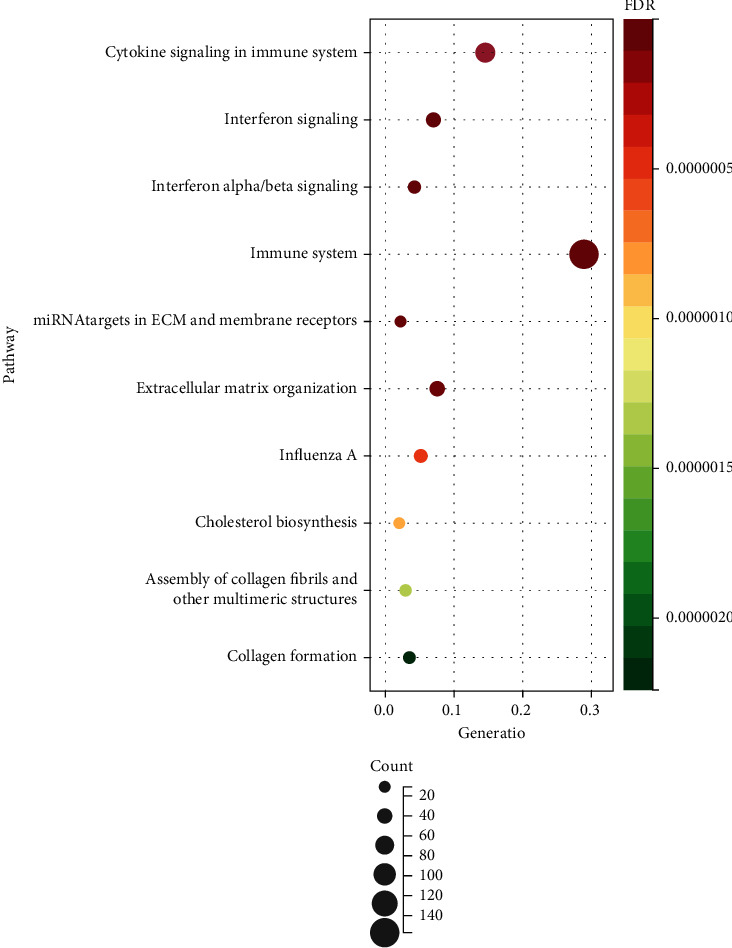
Top 10 significantly enriched KEGG pathway in DEGs. The gene ratio was represented by the *x*-axis, while the pathway name was represented by the *y*-axis. The red color showed that the pathway's FDR value was lower, and the larger the circle, the more genes the pathway contained.

We used WebGestalt to perform GO enrichment analysis on the above DEGs to investigate how DEGs contribute to an organism's biology at the molecular, cellular, and organism levels. The findings are divided into three categories: molecular function (MF), cellular component (CC), and biological process (BP) (Figure 2(a)). The analysis showed significant enrichment in BP including immune response and regulation of response to stress, changes in CC containing endoplasmic reticulum, etc., and the enrichment of MF included several binding and activity related functions.  Figure 2(b) depicts the top ten GO terms with the most significant rich concentration data, and the network may imply a link between these channels. Changes in KEGG pathways were significantly enriched in immune system (cytokine signaling in immune system and interferon signaling etc.). FDR < 0.001 was used as the cut-off criterion for the results (Figure 3).

### 3.3. Construction of PPI Network and Identification and Enrichment of Functional Modules

**Figure 4:**
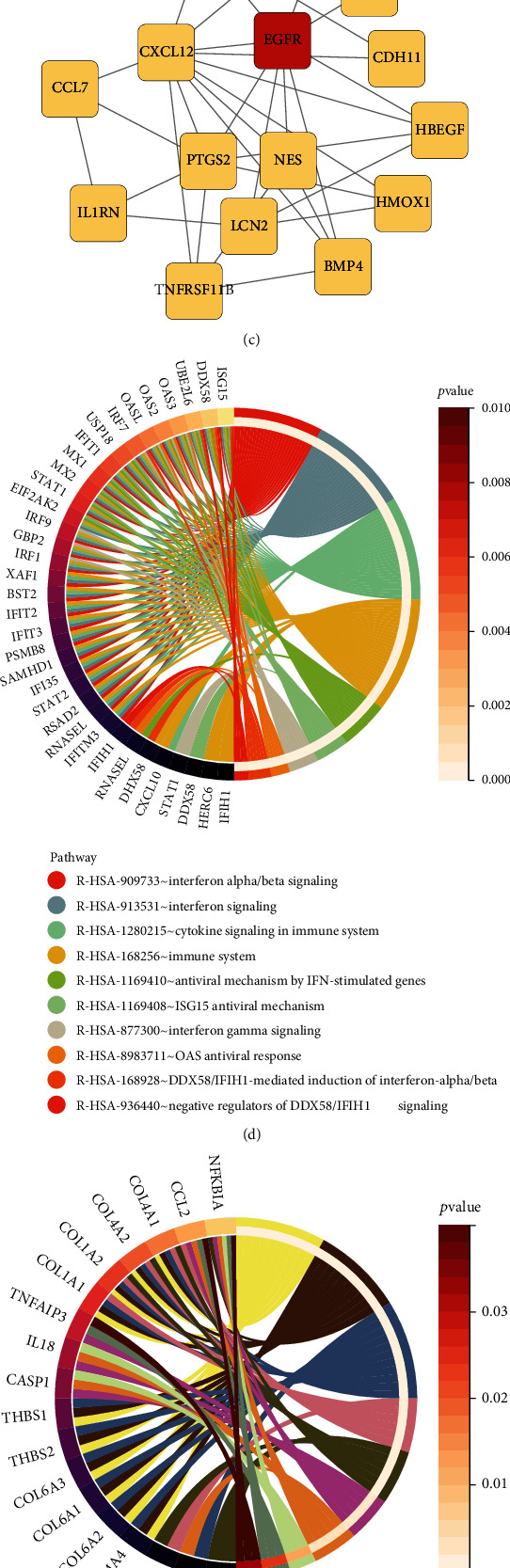
Three modules and enrichment analysis of PPI network. (a) Module 1 was made up of 39 nodes and 677 edges. (b) Module 2 included 31 nodes and 263 edges. (c) Module 3 contained 15 nodes and 33 edges. (d–f) Module enrichment outcomes.

The above DEGs were entered into the STRING platform to create a PPI network, and the results were uploaded to Cytoscape for further study. We found three strongly interacting modules after analyzing MCODE in Cytoscape (degree cut off = 2, node score cut off = 0.2, *k* − core = 2, and max.depth = 100). We enriched the genes in three modules using DAVID to determine the function of these subnetworks (Figure 4). The findings revealed that module 1 was strongly enriched in the interferon signaling route, module 2 was significantly enriched in the ECM-receptor interaction and PIK-Akt signaling pathways, and module 3 was significantly enriched in the EGFR-related pathways.

### 3.4. Identification of Hub Gene and Selection of BICC1-Related ceRNA

**Figure 5:**
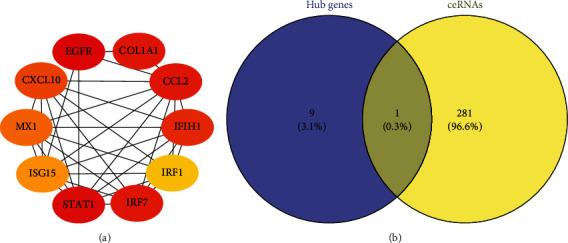
Identification of hub genes and key genes. (a) The subnetwork displayed ten hub genes according to the degree algorithm. (b) The Venn diagram exhibited that the common gene was 0.3% of the total 291 DEGs.

Through topological analysis in cytoHubba, the top ten genes of degree algorithm in the network were identified EGFR, STAT1, IRF7, CCL2, COL1A1, IFIH1, CXCL10, MX1, ISG15, and IRF1 as hub genes (Figure 5(a) and Table 1). BICC1 was entered into starBase v2.0 to assess its involvement in causing aging characteristics, and 282 related ceRNAs were obtained based on multidimensional sequencing data. Nominal *P* < 0.001, FDR < 0.01, and hitMiRnum > 35 were set as cut-off criteria (Supplementary materials Table S1). EGFR was identified as a hub gene and BICC1-related ceRNA (Figure 5(b)).

### 3.5. Construction of TF-Gene Network between BICC1 and EGFR

**Figure 6:**
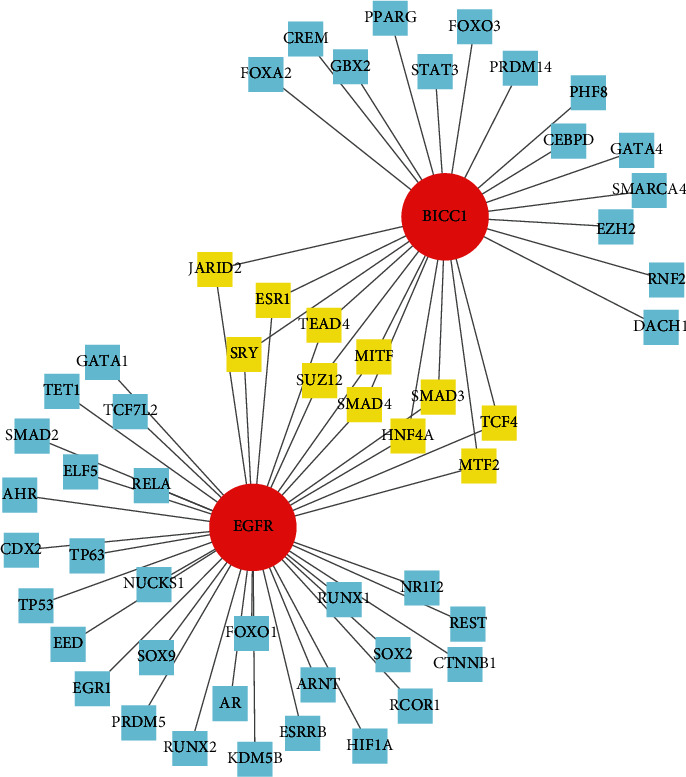
TF-gene interaction network with BICC1 and EGFR. The yellow-highlighted nodes represented common TF-genes between BICC1 and EGFR. The network contained 55 nodes and 64 edges.

NetworkAnalyst was used to collect TF-gene interactions. There were 11 common TF-genes (JARID2, ESR1, SRY, TEAD4, SUZ12, MITF, SMAD4, SMAD3, HNF4A, TCF4, and MTF2) identified. The TF-gene interaction network is depicted in Figure 6.

### 3.6. Construction of Gene-miRNA Network

**Figure 7:**
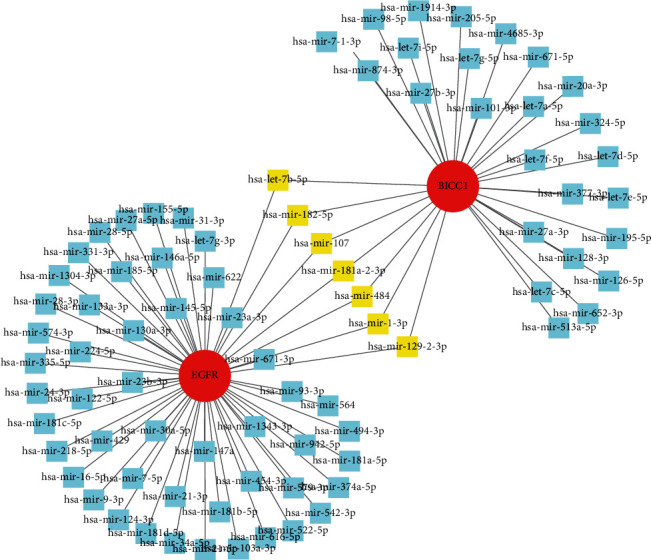
The network showed the TF-miRNA coregulatory network. The highlight yellow color were common miRNAs between BICC1 and EGFR. The network contained 83 nodes and 88 edges, including 81 miRNAs.

We built gene-miRNA networks of BICC1 and EGFR using the TarBase v8.0 database in the NetworkAnalyst platform. The network displayed common seven essential miRNAs (hsa-let-7b-5p, hsa-mir-182-5p, hsa-mir-107, hsa-mir-181a-2-3p, hsa-mir-484, hsa-mir-1-3p, and hsa-mir-129-2-3p) between BICC1 and EGFR (Figure 7).

### 3.7. Comparison of Key Gene Expressions

**Figure 8:**
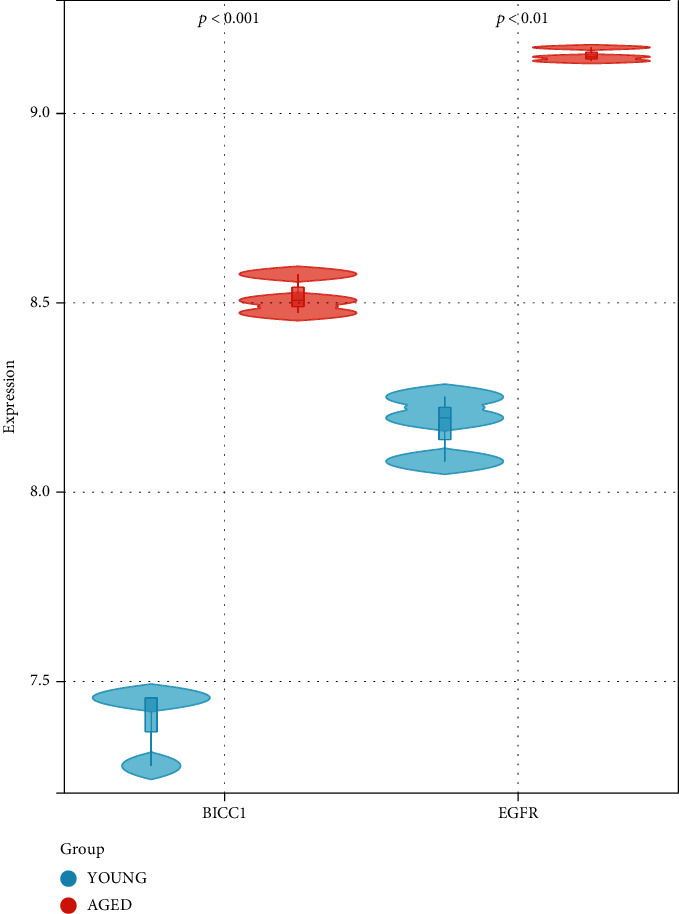
Relative mRNA expression of BICC1 and EGFR in young and aged Sca-1+ BMSCs based on GSE116925.

We submitted the BICC1 and EGFR expression data from GSE116925 to Sangerbox and obtained the violin image (Figure 8). BICC1 and EGFR were significantly upregulated in the aged Sca-1+ BMSCs compared with the young Sca-1+ BMSCs.

## 4. Discussion

Senile osteoporosis is a condition that occurs as a result of a steady loss in systemic bone density caused by aging [25]. Their differentiation potential is critical for maintaining bone metabolic balance. Osteogenic differentiation capacity of BMSCs from human decreased [26–28]. Meanwhile, the senescence-associated secretory phenotype (SASP), which induces and enhances chronic inflammation in a multitude of age-related diseases, may have a role in BMSCS osteogenic differentiation [29]. This results in age-related bone mass loss, which eventually leads to osteoporosis. Therefore, understanding the mechanisms that regulate the aging process and the osteogenic differentiation direction of BMSCs related to age is critical for the treatment of senile osteoporosis.

In recent years, geneticists have identified *Bicc1* as a gene that plays a key role in regulating bone mineral density [30]. However, little research has been conducted to determine whether *Bicc1* plays a role in the osteogenic differentiation of aging BMSCs. Therefore, through the comprehensive analysis of GEO data, we selected the gene expression profiles of Sca-1+ BMSCs from young and aged mice. The GO and KEGG pathway enrichment analyses of differentially expressed genes in the DAVID database revealed that DEGs mostly activated the immune-related signal pathway and the interferon pathway. We examined the PPI network of DEGs and built functional modules. Then, we screened the hub gene in the PPI network and built a prospective BICC1-related miRNA-mRNA network in aged BMSCs.

A GSE116925 dataset including Sca-1+CD29+CD45–CD11b–BMSC gene expression data from 3- and 18-month-old mice was sorted for studies. Stem cell antigen-1 (Sca-1) proteins are biological markers that are found all across stem cells [31]. Some researchers theorize that cells with a low Sca-1 protein expression would naturally differentiate. CD29, as a fibronectin receptor, participates in a number of cell-cell and cell-matrix interactions [32]. It was responsible for a wide range of vital biological tasks, including embryonic development, wound healing, hemostasis, and the prevention of programmed cell death. The expression of CD29 is linked to MSC migration.

Sca-1+ BMSCs have the properties of skeletal stem cells. The development, maintenance, and bone remodeling are jointly maintained by a variety of regional specific bone stem cells (SSCs) [33]. Bone marrow, growth plate, and periosteum all contain SSCs, but their heterogeneity and functional distinctions have not been adequately addressed. According to research, Sca-1+ BMSCs were drawn to bone resorption sites by cytokines generated by osteoclasts during bone resorption in order to finish the process of bone formation [34]. At the initial absorption site, the exposed bone matrix provides a protein-rich microenvironment for the osteogenic differentiation of BMSCs.

Multifunctional growth factors (BMPs, IGF-I, IGF-II, and PDGF) have been released from bone matrix to regulate the differentiation of skeletal stem cells into osteoblasts [35]. Growth factors are produced during osteoclast bone resorption, ensuring that BMSCs do not differentiate into osteoblasts before being recruited into the osteogenic microenvironment [36]. This process ensures that new bone formation always begins at the location of fresh absorption, preserving the mechanical qualities of the bone microstructure. At the present time, most studies focus on the effect of various factors on osteogenesis, but the specific molecular regulation mechanism of the stem cell differentiation process has rarely been reported, which has become the main bottleneck for the clinical application of MSCs in the prevention and treatment of osteoporosis.

We examined the hub gene detection module using the PPI network. Hub genes were defined as those with a high gene interaction rate or degree value, and we identified the top ten hub genes based on degree value (EGFR, STAT1, IRF7, CCL2, COL1A1, IFIH1, CXCL10, MX1, and IRF1). We performed ceRNA analysis of BICC1 using TargetScan and starBase v2.0 to determine the function of BICC1 and DEGs in specific biological processes. The study discovered that BICC1 is an endogenous competitive target gene of EGFR. Recent research has revealed that members of the EGF family play a key role in bone biology [37]. EGF increased BMSC and osteoclast precursor cell proliferation and migration while decreasing differentiation [38]. Stimulating BMSCs with EGFR ligands might increase the production of growth factors and cytokines such as vascular endothelial growth factor (VEGF), platelet-derived growth factor BB (PDGF-B-BB), and interleukin-6 and 8. The RAS/MAPK, PLC/PKC, PI3K/AKT, and STAT signaling pathways can all be activated by the EGFR signaling pathway [39]. In EGFR mutant mice, the EGFR signal also governs the early stage of BMSC proliferation prior to osteogenic differentiation, which is linked to increased bone mass. The EGFR signaling pathway suppresses the production of key osteogenic factors such as Runx2 and Osterix [40]. Furthermore, EGFR signaling can improve osteoprogenitor survival and antiapoptotic effects via activating the transcription factor EGR-2 [41]. Recently, researchers discovered that EGFR signaling in BMSCs improves mechanical transduction, implying that the EGF system functions as a mechanical sensor in BMSCs [42]. Integrins and calcium channels connect mechanical forces in the microenvironment to cell membranes and primary cilia. BICC1 is coexpressed with PKD2, which is encoded in osteoblasts, and it plays a role in osteoblast development [43]. The polycystic protein 2 encoded by Pkd2 is a calcium ion channel protein that is found on the cilia of osteoblasts. Primary cilia containing PKD2/PC2 and PKD1/PC1 operate as osteoporosis-related mechanoreceptors in osteoblasts and renal epithelial cells, and BICC1 has been found in renal cell primary cilia [44]. Researchers also discovered that BICC1 plays a role in osteoblast development by suppressing miR-17 transcription and silencing the Pkd2 gene [45]. Therefore, we speculated whether BICC1 could regulate BMD of the elderly and osteogenic differentiation of BMSCs through the EGFR pathway.

The protein encoded by STAT1 gene is the member of STAT protein family, which is phosphorylated on tyrosine and serine residues and responds to cytokines and growth hormones such as IFN-*α*, IFN-*β*, PDGF, and EGF [46]. After type I interferon (IFN-*α* and IFN-*β*) binds to cell surface receptors, signal transduction via protein kinases activates Jak kinase (TYK2 and JAK1) and phosphorylates tyrosine in STAT1 and STAT2 [47]. IRF7 is a transcriptional regulator of the type I interferon dependent immune response and plays an important role in the innate immune response against DNA and RNA viruses. CXVL10, MX1, IRF1, and IRF7 are all involved in bone immunity and interferon signaling.

BICC1 and EGFR miRNA and transcription factor analyses found 11 transcription factors that could potentially be coregulated. Smad3 and Smad4 are essential members of the SMAD family, and Smad4 is the core molecule of the TGF superfamily signaling mechanism [48]. The TGF-1/smad4 signaling pathway is critical for osteoblast development, differentiation, and death [49]. We discovered seven coregulated BICC1 and EGFR miRNAs, with miR-484 being the ncRNA most closely related to bone density and fracture [50]. We also discovered that the expression of BICC1 and EGFR was higher in the aged Sca-1+ BMSCs than in the young.

## 5. Conclusion

In conclusion, our findings indicated that the immune system and interferon signaling pathways are mostly active in aged Sca-1+ BMSCs. BICC1, a critical gene for bone mineral density, is linked to the hub gene EGFR, and their shared transcription factors and miRNAs have been linked to BMSCs activity. These findings suggested that BICC1 may be important in the regulation of bone density and osteogenic differentiation in elderly osteoporosis. However, there was no experimental evidence for differential gene predictions, such as reverse transcription polymerase chain reaction and western blot. As a result, more research was required to uncover the potential regulatory mechanisms of BICC1 in elderly osteoporosis.

## Figures and Tables

**Table 1:** Topological result exploration for top ten hub genes in PPI of DEGs.

Huh gene	Degree	MNC	EPC	Closeness	Betweenness	Stress
EGFR	107	102	95.239	356.68333	74234.52841	672400
STAT1	91	89	109.877	326.1	24087.0854	308648
IRF7	73	73	106.82	291.9	7334.7781	129380
CCL2	73	73	97.514	324.71667	19443.04594	389412
COL1A1	73	73	81.424	307.58333	13754.01334	218046
IFIH1	67	66	105.53	286.35	3587.98409	63874
CXCL10	66	66	104.772	299.91667	7032.93555	147128
MX1	62	61	103.843	280.51667	3096.12971	58414
ISG15	61	61	102.902	290.9	3610.14843	77540
IRF1	60	60	103.746	283.8	3518.23266	73162

## Data Availability

The data used to support the findings of this study are available from the corresponding author upon reasonable request.

## Supplementary Materials

Supplementary Materials The ceRNA data associated with BICC1.
